# Supplementary figures and images for: Three classes of epigenomic regulators converge to hyperactivate the essential maternal gene deadhead within a heterochromatin mini-domain
Source: PLoS Genet. 2022 Jan 4;18(1):e1009615. doi: 10.1371/journal.pgen.1009615 (PMC8759638; doi:10.1371/journal.pgen.1009615)

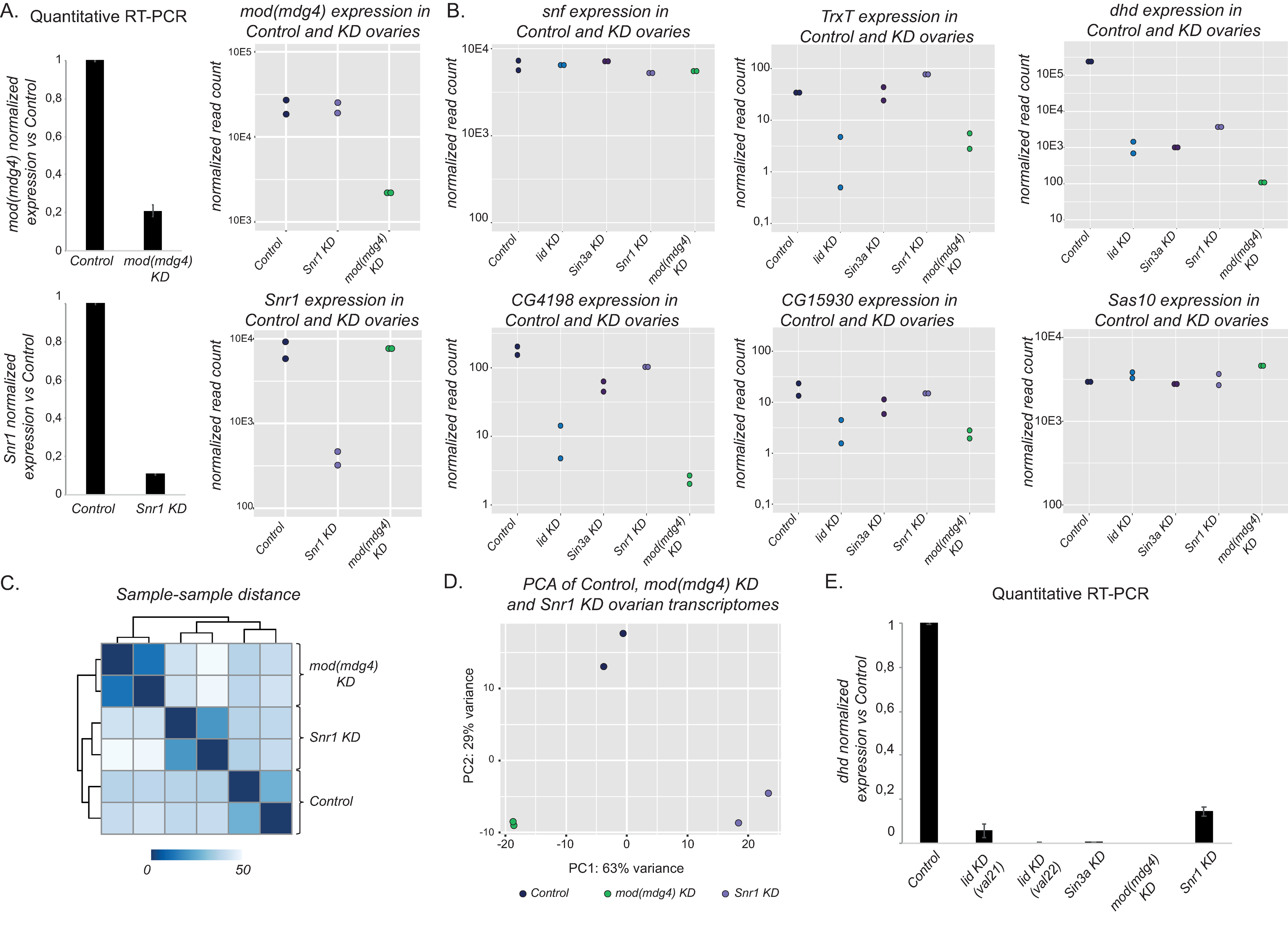

Supplement: S1 Fig — A-mod(mdg4) KD and Snr1 KD are efficient in the female germline. Left: RT-qPCR quantification of mod(mdg4) (top) or Snr1 (bottom) mRNA levels in Control and KD ovaries (normalized to rp49 and relative to expression in Control ovaries). Data from biological duplicates analyzed in technical duplicates are presented as mean ± SEM. Right: Quantification of mod(mdg4) (top) and Snr1(bottom) counts in RNA-seq data from Fig 1B. Both duplicates are shown. B-lid, Sin3a, Snr1 and mod(mdg4) KDs downregulate dhd but do not significantly affect its neighboring genes. Quantification of counts in RNA-seq data for dhd and its neighboring genes in Control, lid, Sin3a, Snr1 and mod(mdg4) KD show that low-expressing genes in the dhd region are not or only modestly impacted by the KDs. Both duplicates are shown. C-Limited overlap in the effects of mod(mdg4) and Snr1 KDs. Hierarchical clustering of sample distance heatmap of RNA-seq samples. D-Principal component analysis for RNA-seq samples. E- lid, Sin3a, mod(mdg4) and Snr1 KD severely downregulate dhd expression. RT-qPCR quantification of dhd mRNA levels in ovaries of indicated genotypes (normalized to rp49 and relative to expression in Control ovaries). Two different shRNA constructs (val21 and val22) against lid were tested. Data from biological duplicates analyzed in technical duplicates are presented as mean ± SEM. (TIF) [file pgen.1009615.s001.tif]

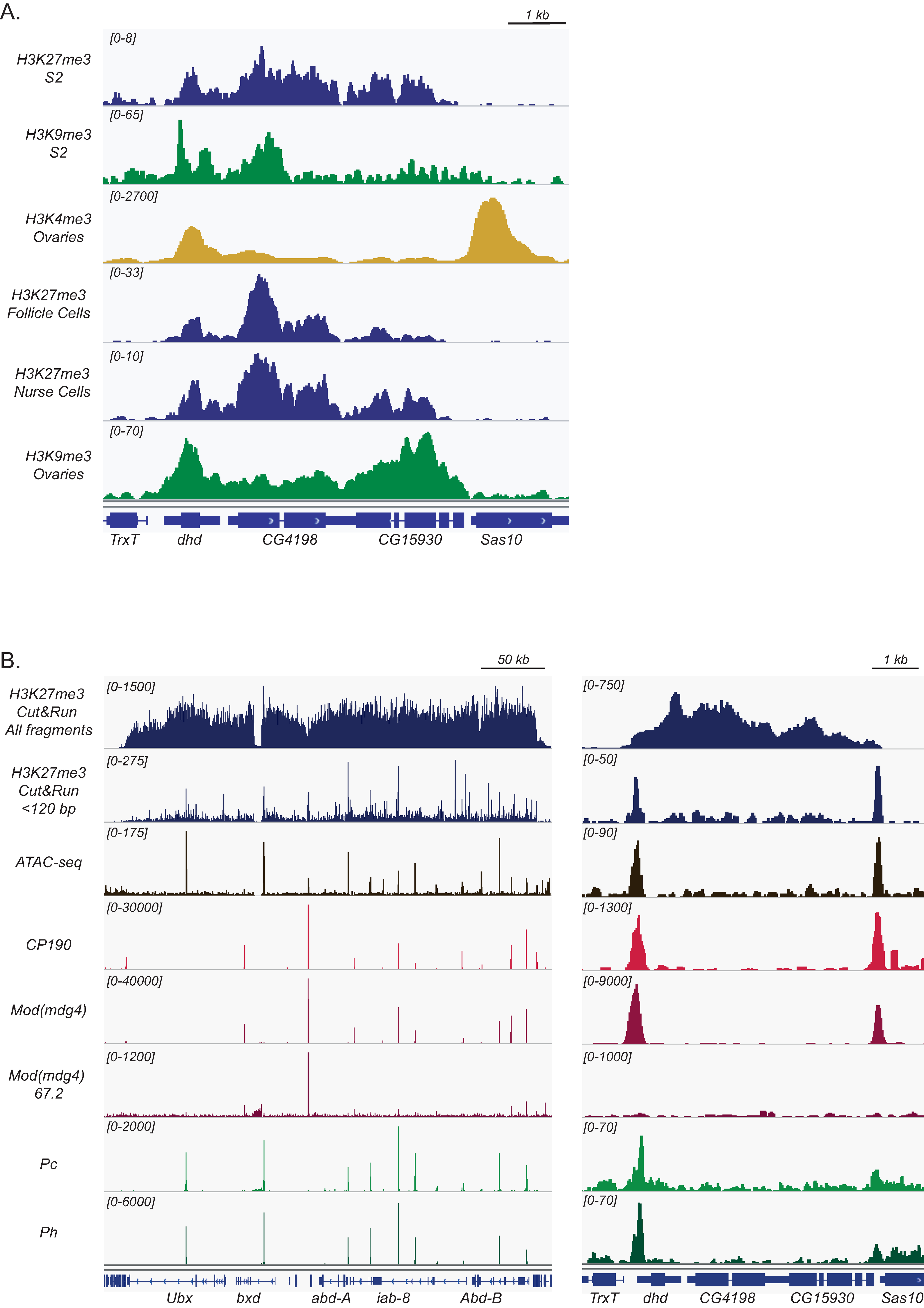

Supplement: S2 Fig — A—Histone modification profiles at the dhd region in cultured embryonic cells and ovaries. ChIP-seq data showing the active mark H3K4me3 (yellow) [5], and the repressive marks H3K9me3 (green) [40,45] and H3K27me3 (blue) (accession number GSE145282, [50]). B—Short fragment peaks align with known regulatory elements. Genome browser views of the bithorax complex (BX-C) (left) and the dhd region (right). Display of H3K27me3 Cut&Run (from Control ovaries, all fragments and <120bp fragments), ATAC-seq (from S2 cells, [35]), CP190 ChIP-seq (from Kc cells, [38]), Mod(mdg4) (all isoforms) and Mod(mdg4)67.2 isoform ChIP-seq (from Kc cells, [24]) Polycomb (Pc) and Polyhomeotic (Ph) ChIP-seq (from S2 cells, [37]). Cut&Run short fragments largely overlap with peaks from the other tracks displayed. (TIF) [file pgen.1009615.s002.tif]

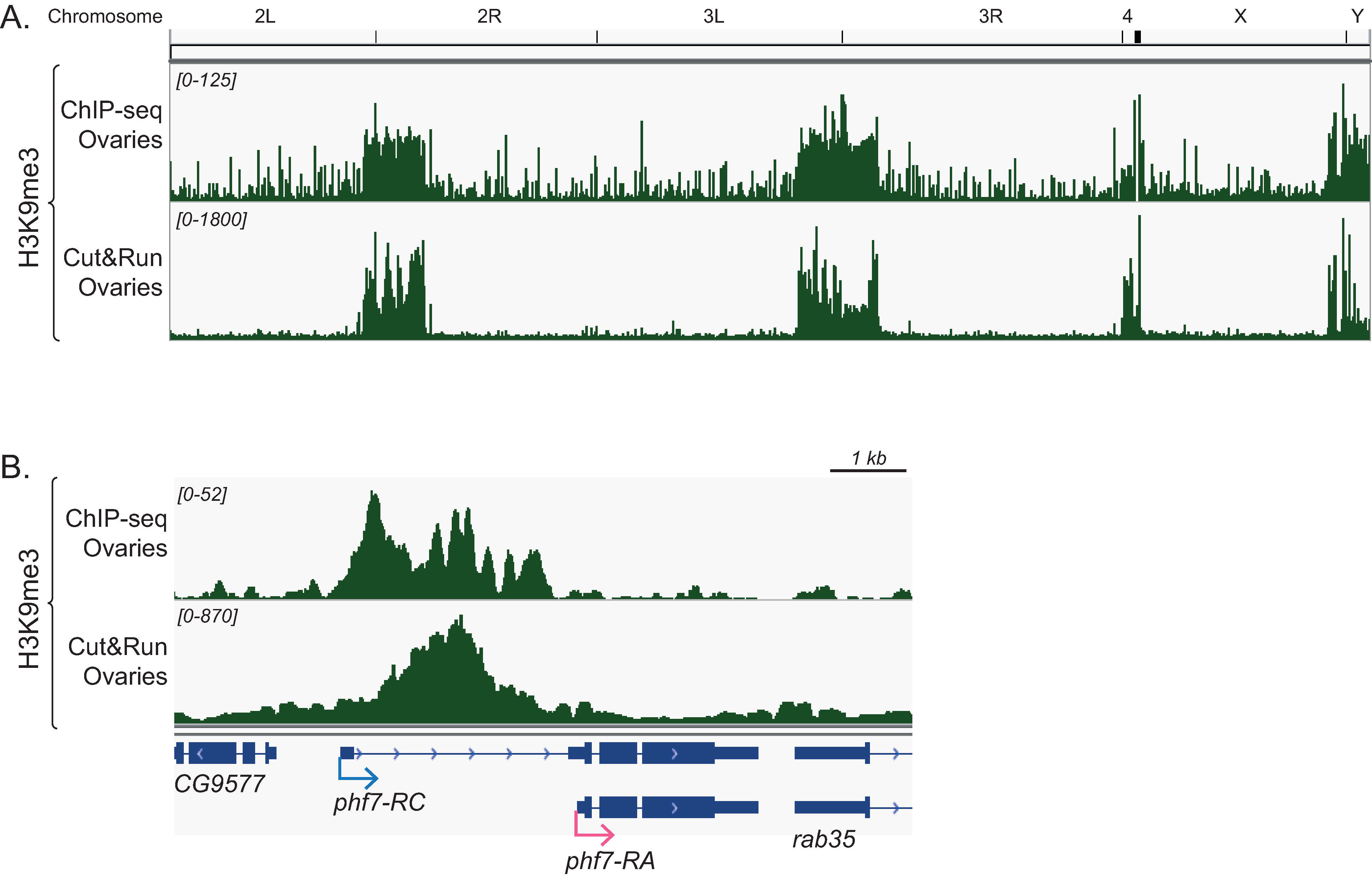

Supplement: S3 Fig — A- Cut&Run shows the expected enrichment of H3K9me3 at pericentromeric heterochromatin. Genome browser views of H3K9me3 ChIP-seq [45] and Cut&Run signal in all chromosomes. B- Cut&Run detects a previously identified H3K9me3 peak over a testis-specific TSS [45]. Genome browser view of phf7 and neighboring genes. Blue arrow indicates testis-specific TSS and magenta arrow indicates ovary-specific TSS. (TIF) [file pgen.1009615.s003.tif]

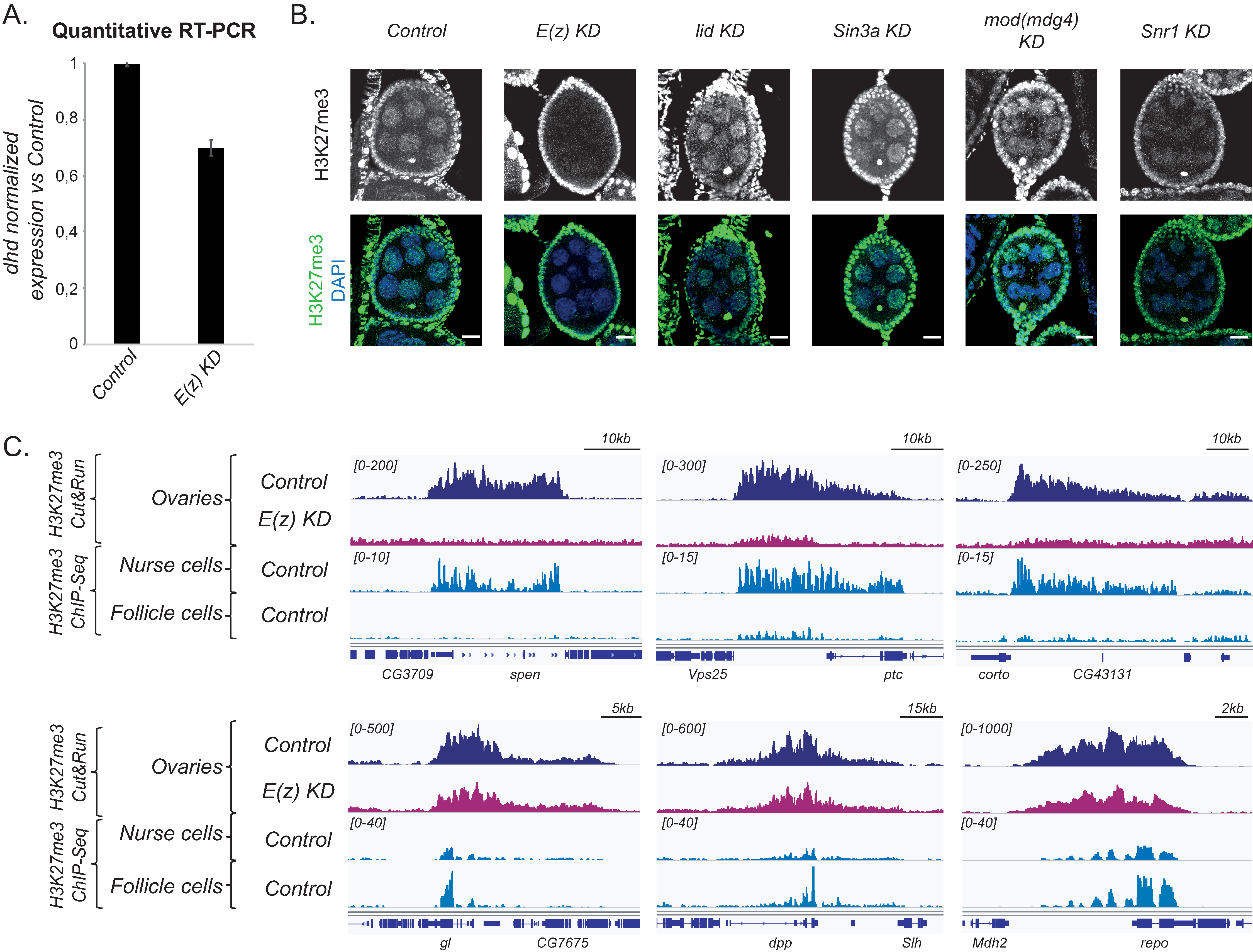

Supplement: S4 Fig — A-E(z) KD does not severely affect dhd expression. RT-qPCR quantification of dhd mRNA levels in Control and E(z) KD ovaries (normalized to rp49 and relative to expression in Control ovaries). Data from biological duplicates analyzed in technical duplicates are presented as mean ± SEM. B- E(z) KD and Snr1 KD affect H3K27me3 levels in nurse cells. Confocal images of representative egg chambers in Control, E(z) KD, lid KD, Sin3a KD, mod(mdg4) KD and Snr1 KD. In control ovaries, H3K27me3 staining marks somatic follicle cell nuclei, the karyosome and germline nurse cell nuclei. In E(z) KD ovaries the karyosome and nurse cells loose staining of the histone mark but follicle cells are marked normally. No notable change is observed in lid KD, Sin3a KD or mod(mdg4) KD while in Snr1 KD nurse cells staining is less intense. Scale bar 10μm. C- Cut&Run in whole ovaries captures signal from both somatic and germline cells. Genome browser views of H3K27me3 Cut&Run signal in Control and E(z) KD ovaries and H3K27me3 ChIP-seq from FACS sorted nurse cells and somatic follicle cells [50]. Upper panels show representative loci enriched for the mark solely in nurse cells (germline) and absent in E(z) KD ovaries. Lower panels show H3K27me3 domains where the signal comes almost exclusively from follicle cells and is not significantly affected in the germline E(z) KD. (TIF) [file pgen.1009615.s004.tif]

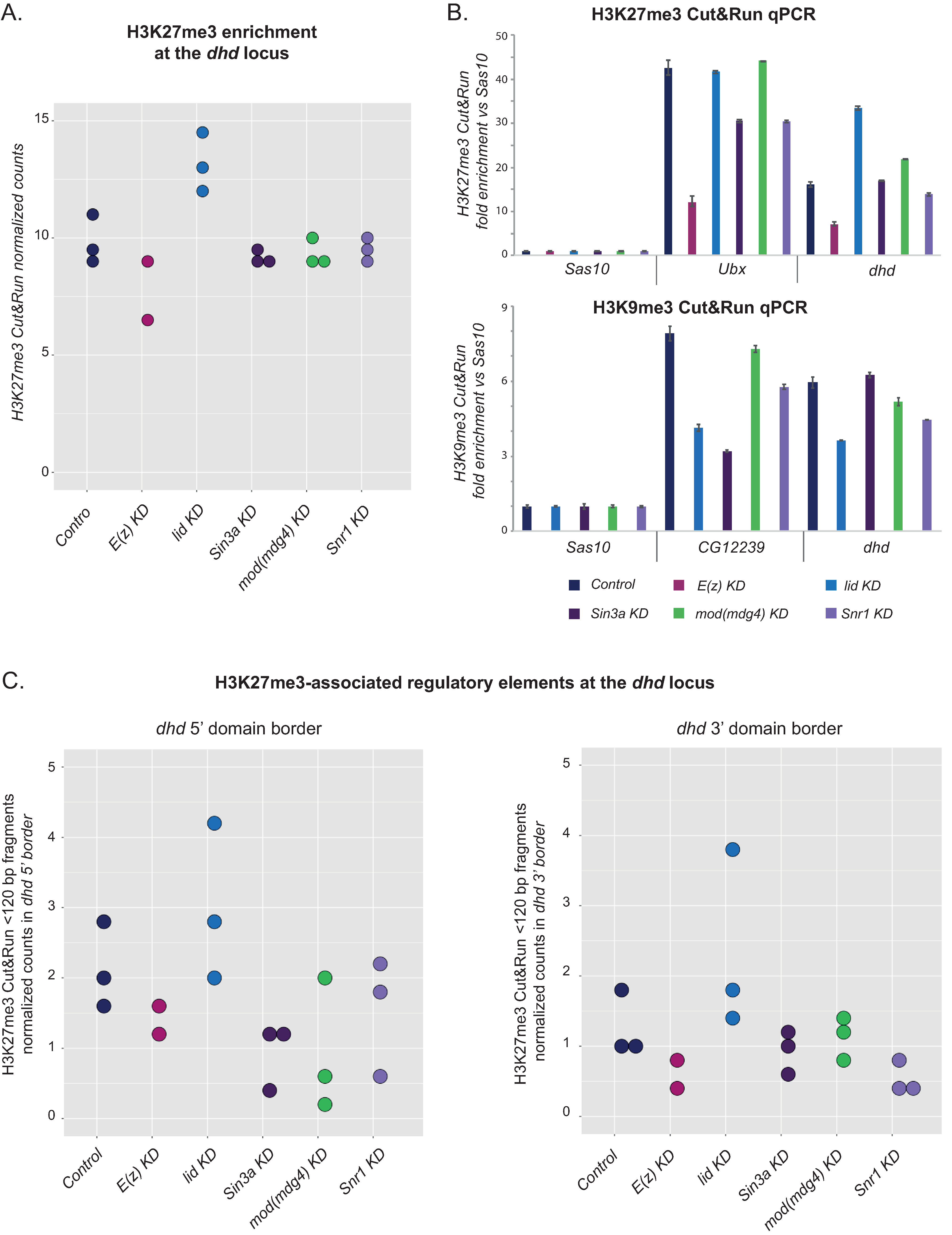

Supplement: S5 Fig — A—H3K27me3 Cut&Run signal at the dhd locus from Control and KD ovaries. Left: Dotplot showing normalized read counts of H3K27me3 Cut&Run at the dhd domain from independent biological triplicates of the indicated genotypes (duplicates for E(z) KD). B—Cut&Run qPCR yields reproducible data among replicates. Biological replicates from H3K27me3 and H3K9me3 Cut&Run-qPCR in Control and KD ovaries shown in Fig 3E. The Sas10 gene was used as negative control and Ubx and CG12239 as positive controls for H3K27me3 and H3K9me3 respectively. Fold enrichment was calculated relative to Sas10. Error bars show technical variability. C—Sin3a KD, Snr1 KD and mod(mdg4) KD affect the stability of the H3K27me3-associated regulatory elements at the dhd mini-domain. Dotplot showing normalized read counts of H3K27me3 Cut&Run <120bp fragments at dhd regulatory elements from independent biological triplicates of the indicated genotypes (duplicates for E(z) KD). 5’ and 3’ border elements are plotted separately. (TIF) [file pgen.1009615.s005.tif]

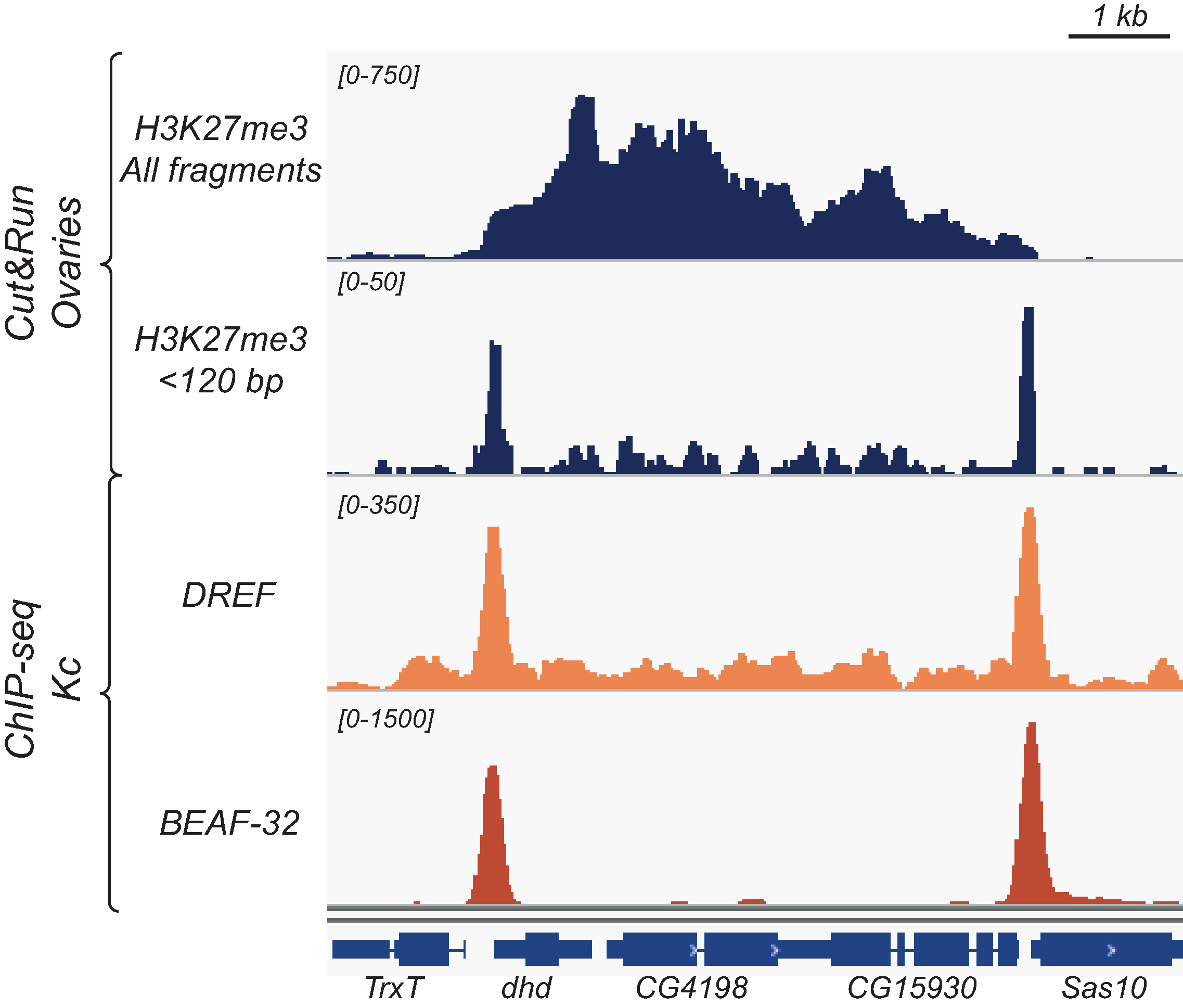

Supplement: S6 Fig — Genome browser views of ovarian H3K27me3 Cut&Run (all fragments and <120bp fragments) and Dref and Beaf-32 ChIP-seq (from Kc cells, [38]). <120 bp fragment peaks at the dhd domain borders align with DREF and Beaf-32 peaks. (TIF) [file pgen.1009615.s006.tif]

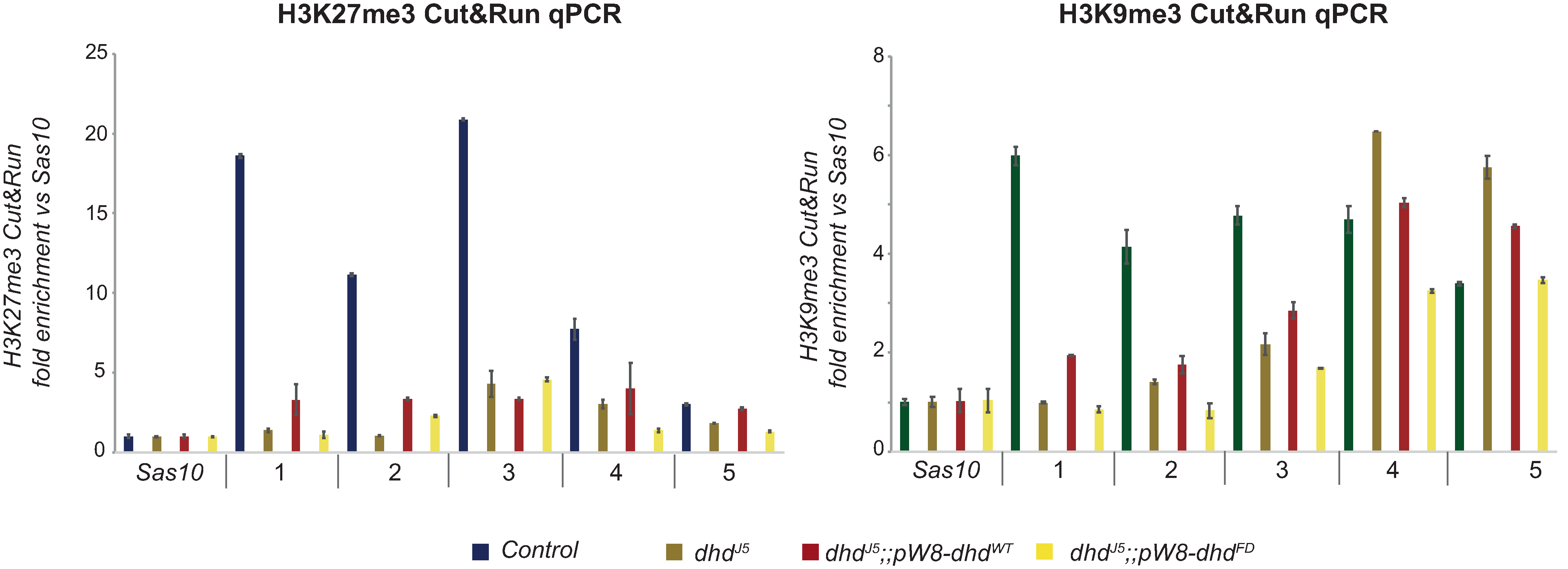

Supplement: S7 Fig — Biological replicates of H3K27me3 and H3K9me3 Cut&Run-qPCR in Control, dhdJ5, dhdJ5;;pW8-dhdWT and dhdJ5;;pW8-dhdFD ovaries shown in Fig 5D. Fold enrichment was calculated relative to Sas10. Error bars show technical variability. (TIF) [file pgen.1009615.s007.tif]

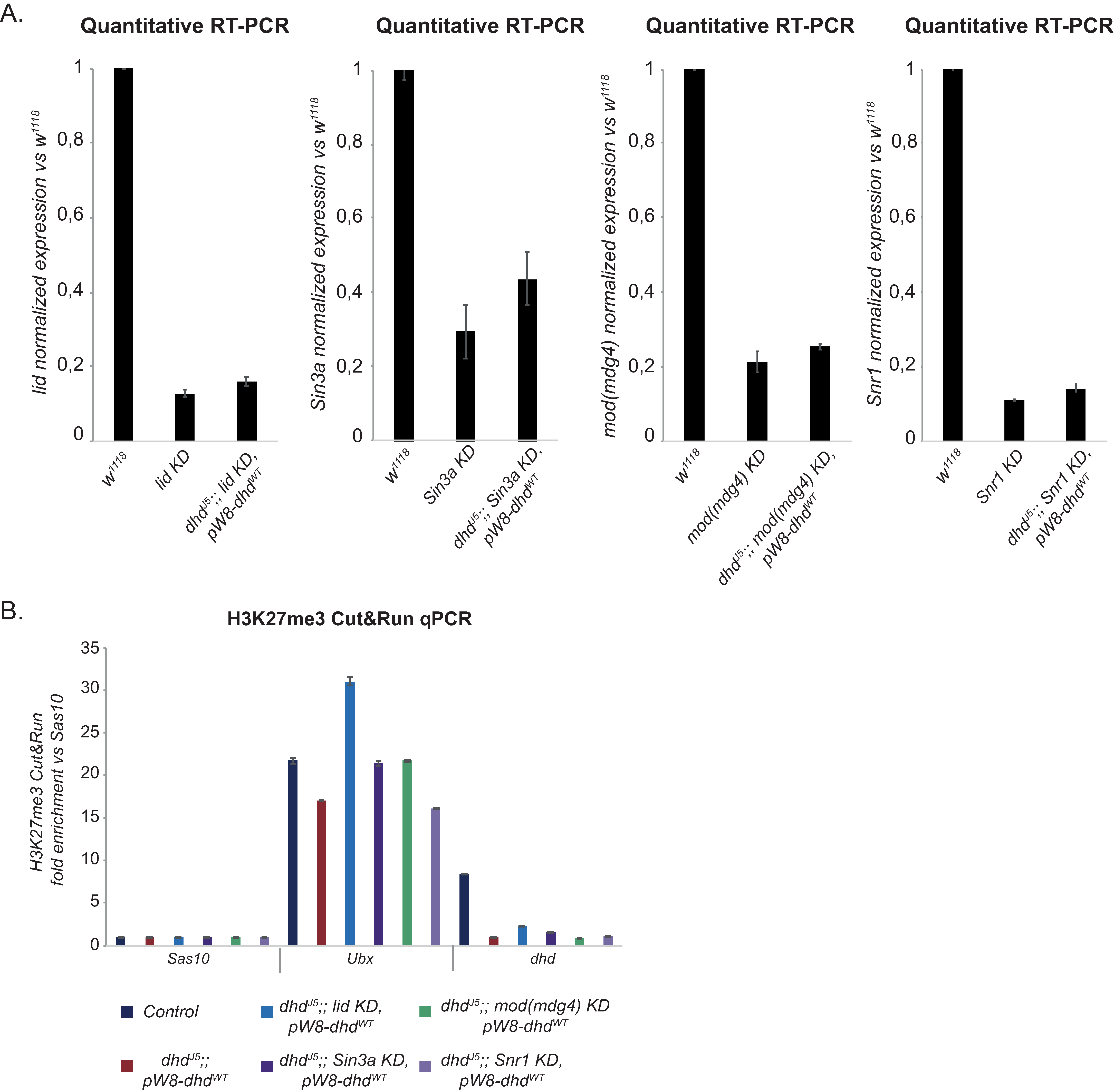

Supplement: S8 Fig — A—lid KD, Sin3a KD, mod(mdg4) KD and Snr1 KD are efficient in the female germline of rescue flies. From left to right: RT-qPCR quantification of lid, Sin3a, mod(mdg4) and Snr1 mRNA levels in ovaries of the indicated genotypes (normalized to rp49 and relative to expression in w1118 ovaries). Data from biological duplicates analyzed in technical duplicates are presented as mean ± SEM. B—The dhd rescue transgene does not restore H3K27me3 in KD flies. Biological replicates of H3K27me3 Cut&Run-qPCR in the indicated genotypes shown in Fig 6C. The Sas10 gene was used as negative control and Ubx as positive control. Fold enrichment was calculated relative to Sas10. Error bars show technical variability. (TIF) [file pgen.1009615.s008.tif]
