# Supplementary material for: Three classes of epigenomic regulators converge to hyperactivate the essential maternal gene deadhead within a heterochromatin mini-domain
Source: PLoS Genet. 2022 Jan 4;18(1):e1009615. doi: 10.1371/journal.pgen.1009615 (PMC8759638; doi:10.1371/journal.pgen.1009615)
Supplement: S1 Table — (PDF) [file pgen.1009615.s009.pdf]

**Table S1. List of primers used in this paper.**

| Primer | Sequence (5' —> 3') | Purpose |
|---|---|---|
| dhd-for | TCTATGCGACATGGTGTGGT | RT-qPCR |
| dhd-rev | TCCACATCGATCTTGAGCAC | RT-qPCR |
| lid-for | ATTGGTTTCACGAGGATTGC | RT-qPCR |
| lid-rev | CATAGCCACTTGGGTCGATT | RT-qPCR |
| Sin3a-for | CGACAAATGGGTATCGTTCC | RT-qPCR |
| Sin3a-rev | GACCAGGTCCAGCTCGAAT | RT-qPCR |
| mod(mdg4)-for | CAACAGATCACCGTGCAAAC | RT-qPCR |
| mod(mdg4)-rev | GTTCAGATTTCGTGGGCAAT | RT-qPCR |
| Snr1-for | TCAGCTCCCACATCTTAGCC | RT-qPCR |
| Snr1-rev | ACTGGCGTATCGGAAGTGTT | RT-qPCR |
| Rp49-for | AAGATCGTGAAGAAGCGCAC | RT-qPCR |
| Rp49-rev | GATACTGTCCCTTGAAGCGG | RT-qPCR |
| Ubx-for | AAAATTCCTCGGCCTGATTC | Cut&Run-qPCR |
| Ubx-rev | AAAAATGGGCGTAGCTCAGA | Cut&Run-qPCR |
| Sas10-for | AGGAGGAGCAGCAGGATGT | Cut&Run-qPCR |
| Sas10-rev | AGATCGCGGTCATCGTCTT | Cut&Run-qPCR |
| CG12239-for | AGATGAGGGACGAAGGATTG | Cut&Run-qPCR |
| CG12239-rev | TTCCTCGGTACGTTCAGCTT | Cut&Run-qPCR |
| dhd-3UTR-for | GTTTAGCTTGTAAGCGCGAGA | Cut&Run-qPCR |
| dhd-3UTR-rev | ATATCATCTGGTCACTGCTGTTG | Cut&Run-qPCR |
| CG4198-1-for | GAGCAAGAAGAATGGCCAAC | Cut&Run-qPCR |
| CG4198-1-rev | CGATCGTTGAACTCCTGGAT | Cut&Run-qPCR |
| CG4198-2-for | GTGTCCATAGCGCAGCAG | Cut&Run-qPCR |
| CG4198-2-for | AAGGTGAACATAACCCCACAA | Cut&Run-qPCR |
| CG15930-1-for | ACCTGGACATCGGCTACATC | Cut&Run-qPCR |
| CG15930-1-rev | ACCATGTGCGAATTTTCGAT | Cut&Run-qPCR |

| | | |
|---|---|---|
| CG15930-2-for | TTATTCCGCATTTTGGCACT | Cut&Run-qPCR |
| CG15930-2-for | GGAAGAAGCCGAGGATAACA | Cut&Run-qPCR |
| ΔDRE-1-for | GAGCAGCAGCCGAATTCGGTACCCCCATATCCCTCCCATATCC | ΔDRE-transgene |
| ΔDRE-1-rev | CACGGCAGGTCTGAATATTATTATCGTAATTATGGCAAAATAATGGC | ΔDRE-transgene |
| ΔDRE-2-for | GATAATAATATTCAGACCTGCCGTGGTGAATAAG | ΔDRE-transgene |
| ΔDRE-2-rev | CACAGGGATGCCACCCGGGATCCGCTAATGGAATCGCAATCGT | ΔDRE-transgene |
| FD-for | GATTGCGATTCCATTAGCGAATGCGGACGATATGCCAACGC | FD-transgene |
| FD-rev | GTCACAGGGATGCCACCCGGGATCCACAAAGAGAAAAACTGTTGTAA | FD-transgene |
