# Supplementary material for: Three classes of epigenomic regulators converge to hyperactivate the essential maternal gene deadhead within a heterochromatin mini-domain
Source: PLoS Genet. 2022 Jan 4;18(1):e1009615. doi: 10.1371/journal.pgen.1009615 (PMC8759638; doi:10.1371/journal.pgen.1009615)
Supplement: S2 Table — (PDF) [file pgen.1009615.s010.pdf]

Table S2. List of antibodies used in this paper.

Antibody	Host animal	Dilution	Experiment	Company (Catalog#)
Anti-Histones	mouse, monoclonal	1:1000	Immunofluorescence	Merck (#F152.C25.WJJ)
Anti-GFP	mouse, monoclonal	1:200	Immunofluorescence	Roche (#118144600001)
Anti-H3K27me3	rabbit, polyclonal	1:500	Immunofluorescence	Merck (#07-449)
Anti-H3K27me3	rabbit, monoclonal	1:100	Cut&Run	Cell Signalling Technology (#9733)
Anti-H3K9me3	rabbit, polyclonal	1:50	Cut&Run	Abcam (#8898)
Anti-DHD	rabbit, polyclonal	1:1000	Western Blot	*Tirmarche et al., 2016 [1]*
Anti-α-tubulin	mouse, monoclonal	1:500	Western Blot	Merck (#T9026)

Reference:

1. Tirmarche S, Kimura S, Dubruille R, Horard B, Loppin B. Unlocking sperm chromatin at fertilization requires a dedicated egg thioredoxin in Drosophila. Nat Commun. 2016;7: 13539. doi: 10.1038/ncomms13539
